# Multiple mobile operating tables for eye surgery

**Published:** 2010-09

**Authors:** Jonathan Pons

**Affiliations:** Ophthalmologist and Programme Director, Good Shepherd Hospital Eye Care Project, P0 Box 218, Siteki, Swaziland. Email: jono@goodshepherdhosp.org

**Figure:**
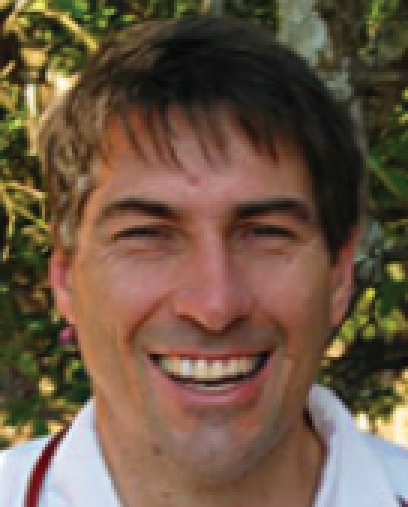


High-volume eye surgery requires that patients be moved quickly in and out of the operating room (OR). Static operating tables in the OR make this difficult. Better patient flow can be achieved when using mobile tables, which can be expensive. We have developed an economical, wheeled operating table that can be constructed in a local engineering workshop.

Because this mobile operating table can transport the patient between the different areas before, during, and after an operation, the patient can stay on the table throughout and does not have to transfer beds. Four tables are in use at any one time: one for a patient being prepared for surgery, one for a patient being given local anaesthetic, one for a patient being operated on, and one for a patient being wheeled out of the OR and returned to the ward.

**Figure:**
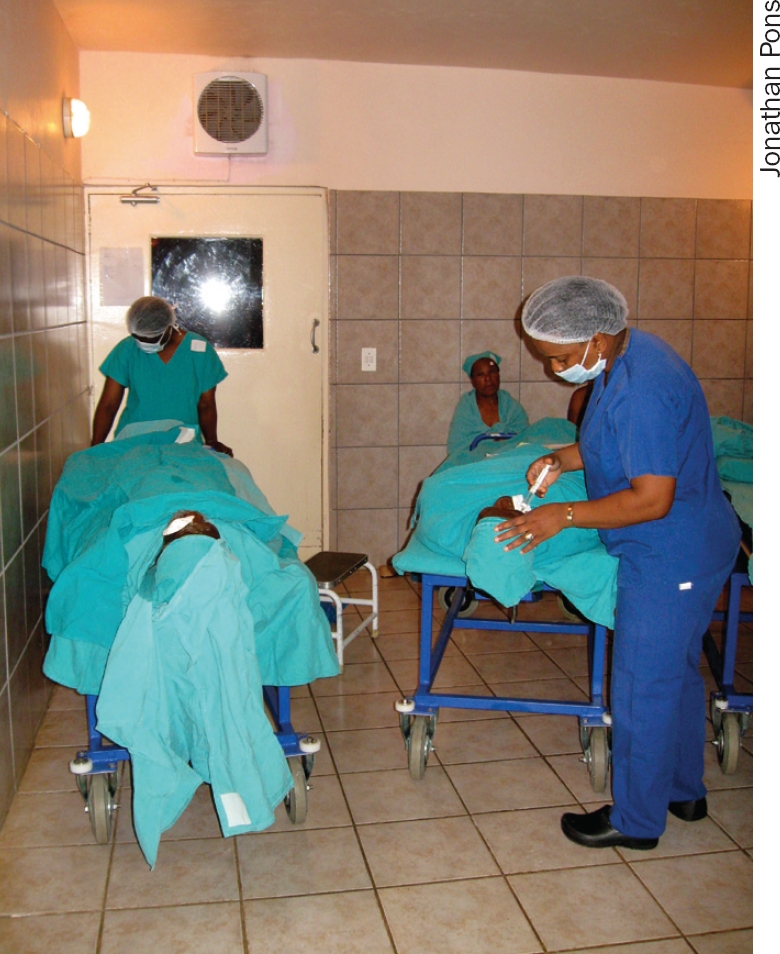
One patient recovers after the operation while another is given local anaesthetic. SWAZILAND

Jonathan Pons

Our finding is that multiple mobile operating tables minimise interruptions in the flow of work; surgeons can concentrate on surgery and may stay scrubbed for the entire day. The tables improve patient comfort through better head support and are also more comfortable for surgeons as they leave more room for surgeons' legs. Another advantage is that fewer staff are needed to manage the flow of patients. The results are dramatically improved time and cost savings: one surgeon can comfortably perform up to forty eye operations in a day.

The tables are designed to be manufactured in a local engineering workshop and the simplified design incorporates the following:

- A tubular steel welded frame
- A bed made of a shaped stainless steel sheet
- Adjustable bed height (using a simple hand-operated thread)
- A head end with non-castoring wheels for stability during surgery
- A foot end with lockable castoring wheels
- Bumpers on all wheel mountings, which prevent contact damage with door frames.

Each table costs approximately UK£300 to produce and drawings are available from the author.

